# Effect of Phosphorus Slag Admixture on the Properties and Hydration Mechanism of Circulating Fluidized Bed Fly Ash-Based Multi-Solid Waste Cementitious Material

**DOI:** 10.3390/ma15196774

**Published:** 2022-09-29

**Authors:** Wei Zhang, Chao Wei, Xiaoming Liu, Zengqi Zhang

**Affiliations:** 1 State Key Laboratory of Advanced Metallurgy, University of Science and Technology, Beijing 100083, China; 2 School of Metallurgical and Ecological Engineering, University of Science and Technology Beijing, Beijing 100083, China

**Keywords:** circulating fluidized bed fly ash, phosphorus slag, admixture, consolidation, cementitious materials

## Abstract

This research aims to reveal the effect of phosphorus slag (PS) admixtures on the properties and hydration mechanism of circulating fluidized bed fly ash (CFA)-based multi-solid waste cementitious material (CWM). The results indicate that PS as an admixture is more helpful for improving the performance of CWM systems compared with blast furnace slag with a high specific surface area (HBFS) and gasification slag (GS). In this work, CWM2 is prepared with 30 wt.% CFA, 10 wt.% red mud (RM), 20 wt.% blast furnace slag (BFS), 10 wt.% PS, and 30 wt.% cement clinker (CC). The compressive strength and expansion value of CWM2 are the optimal (51.15 MPa and 0.70 mm) when the mass ratio of (Ca + Na)/(Si + Al) is 0.84, which can meet the requirements of 42.5 fly ash Portland cement. In addition, the polymerization degree of CWM2-28 days is the optimum (51.57%) because [PO_4_] and [SiO_4_] combine to improve its polymerization structure. The main hydration products are C-S-H gel, C/N-A-S-H gel, and ettringite in CWM, which are conducive to improve the compactness of the micromorphology. In addition, the consolidation of Na, As, Cd, and Hg is promoted in CWM2 by physical encapsulation and charge balance, which meet the drinking water requirements of the World Health Organization (WHO). Therefore, this work provides a new idea for the application of PS as an admixture in CFA-based multi-solid waste cementitious material.

## 1. Introduction

Phosphorus slag (PS) is the granular industrial waste discharged during the production of yellow phosphorus, and approximately 10 tons of PS are discharged when each ton of yellow phosphorus is produced [1]. Recently, the accumulated amount of PS in China has been more than 8 million tons, but its comprehensive utilization rate is low [2]. Most PS is stacked as waste residue with the rapid development of the phosphate industry, which not only occupies land but also harms the ecological environment [3]. Therefore, it is significant to research the cyclic utilization of PS and the development of an environmentally friendly society. Previous scholars have provided many contributions to strengthen the recycling of PS. For instance, the valuable components in PS are efficiently recovered, and calcium carbonate is prepared by carbonization of PS [4,5]. In addition, luminescent glass is developed by high added-value utilization of PS, and the modifier of asphalt concrete is created by PS [6,7]. The recycling of PS has been promoted from the above work, but these studies focus only on high value-added utilization of PS, whereas the comprehensive utilization of PS is still low.

At present, it is found that PS is used as an admixture for multi-solid waste-based cementitious material, which is beneficial for increasing the total dosage of PS and other solid wastes. The strength, microstructure densification, filling effect, and carbon fixation level of cementitious materials are optimal under the action of PS admixture when the mass ratio of total solid waste is less than 30 wt.% [8,9]. The hydration degree and pore structure of cementitious materials are excellent under the action of PS, as the total proportion of solid waste is less than 30 wt.% in cement [10]. It can be seen from the above research that the utilization of various solid wastes is promoted in cementitious materials based on action of PS admixture, but the proportion of solid waste in cementitious materials is less than 30 wt.%.

Circulating fluidized bed fly ash (CFA) is a solid waste containing active Si-Al in power plants, and it has the risk of polluting the environment [11,12]. The average annual emission of CFA has exceeded 280 million tons in China [13,14]. The preparation of cementitious materials is an effective method to improve the utilization of CFA. However, the volume of cementitious materials containing many CFAs is unstable due to the existence of unstable components (f-CaO and SO_3_) in CFA [15], which leads to a utilization of CFA of less than 25% [16,17,18]. Then, relevant scholars found that cementitious materials were prepared by the synergy of CFA and other solid wastes, which not only ameliorates its performance but also improves the utilization of CFA [19,20,21]. Thus, PS could be used as an admixture of CFA-based multi-solid waste cementitious material (CWM) to improve its performance and utilization of CFA. However, PS as an admixture of CWM has not been studied at present. In particular, the effect mechanism of PS on CWM system is missing. Therefore, an effective proposal is proposed presented in this work to fill this research gap.

In this work, CFA-based multi-solid waste cementitious material (CWM) is prepared using CFA, Bayer red mud (RM), blast furnace slag (BFS), and an admixture (blast furnace slag with high specific surface area (HBFS)/phosphorus slag (PS)/gasification slag (GS)). The total dosage of solid waste is increased to 70 wt.% in CWM under the action of the PS admixture. The performance value of CWM has a certain surplus coefficient to overcome the difficulties in the recovery of these solid wastes, such as changing the chemical composition of the solid waste. The compressive strength, volume stability, setting time, and environmental performance in the three CWMs are compared based on different admixtures. More importantly, the action mechanism of the PS admixture on the CWM system is discussed in detail. Therefore, this work is expected to provide a new way for PS admixtures to promote the utilization of CFA, RM, and BFS in cementitious materials.

## 2. Materials and Methods

### 2.1. Physicochemical Performances of Raw Materials

#### 2.1.1. Source of Raw Materials

The raw materials of circulating fluidized bed fly ash (CFA)-based multi-solid waste cementitious material (CWM) are CFA, Bayer red mud (RM), blast furnace slag (BFS), cement clinker (CC), and an admixture. The admixtures are blast furnace slag with a high specific surface area (HBFS), phosphorus slag (PS), and gasification slag (GS). CFA and RM were by-products from a power plant and an aluminum plant, respectively, in Yangquan City, China. BFS was purchased from ironmaking plant in Gongyi City, China. CC is produced from a cement plant in Hebei Province, China. HBFS comes from a steel plant in Hejin City, Shanxi Province, China. PS and GS are provided from a phosphorus plant in Guizhou and a coal gasification plant in Inner Mongolia, China, respectively.

#### 2.1.2. Chemical Compositions

The chemical components of the raw materials were determined by XRF (Shimazu Company, Kyoto, Japan). The atoms in the raw materials are excited by primary X-ray photons, and then secondary characteristic X-ray fluorescence is generated to analyze the chemical composition of the raw materials. The oxide compositions of the CFA, RM, BFS, CC, and an admixture (HBFS, PS, and GS) are shown in Table 1. The main chemical components of CFA are T-CaO (CaO), SiO_2_, Al_2_O_3_, SO_3_, f-CaO, and Fe_2_O_3_. The chemical components of RM are CaO, SiO_2_, Al_2_O_3_, Fe_2_O_3_, and Na_2_O. CC is the main raw material for preparing cement, and its chemical components are CaO, SiO_2_, Al_2_O_3_, and Fe_2_O_3_. The main oxides of BFS, HBFS, PS, and GS include total CaO, SiO_2_ and Al_2_O_3_, which promote the secondary hydration reaction of the CWM system. The loss on ignition (LOI) of these raw materials was tested at 800 °C for 4 h. According to the data comparison in Table 1, the LOI of CFA is 10.38 wt.% higher than that of the other raw materials. CFA contains unburned carbon due to the combustion characteristics of circulating fluidized bed boilers at low temperatures (850–900 °C).

#### 2.1.3. Phase Composition

**Figure 1:**
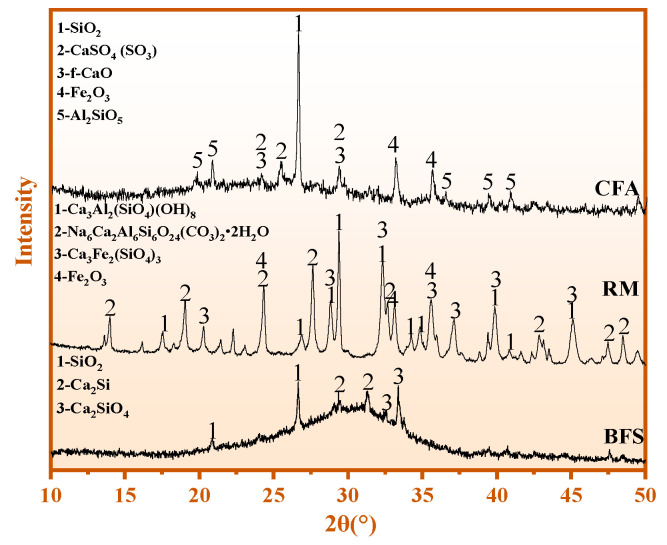
XRD results of RM, CFA, and BFS.

The mineral composition of CFA, RM, and BFS is shown in Figure 1, in which the main phases of CFA are active Si-Al, quartz (SiO_2_), anhydrite (CaSO_4_(SO_3_)), hematite (Fe_2_O_3_), free-calcium oxide (f-CaO), and kyanite (Al_2_SiO_5_). The minerals of RM are c katoite (Ca_3_Al_2_(SiO_4_)(OH)_8_), cancrinite (Na_6_Ca_2_Al_6_Si_6_O_24_(CO_3_)_2_**•**2H_2_O), andradite (Ca_3_Fe_2_(SiO_4_)_3_), and hematite (Fe_2_O_3_). The main phases of BFS are active Si-Al, calcium silicon (Ca_2_Si), dicalcium silicate (Ca_2_SiO_4_), and SiO_2_.

**Figure 2:**
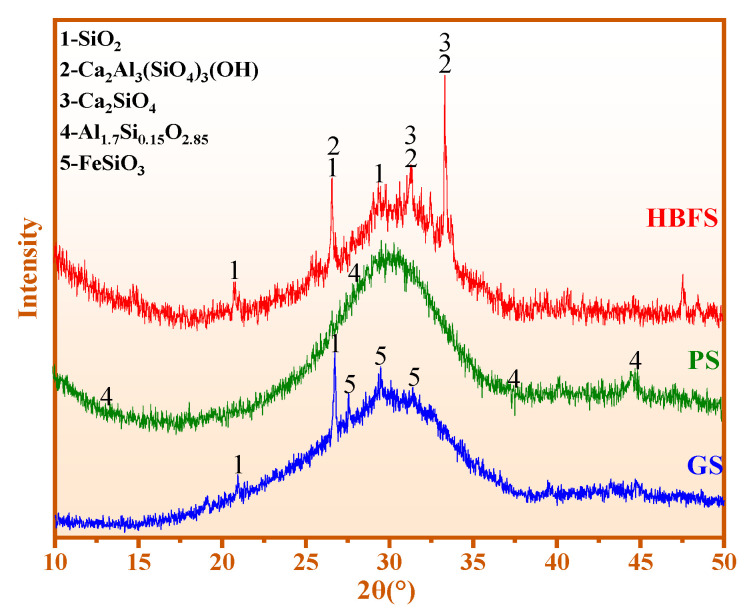
XRD results of HBFS, PS, and GS.

Figure 2 shows the XRD results of three admixtures (HBFS, PS, and GS). The main minerals of HBFS are active Si-Al, quartz (SiO_2_), calcium silicate (Ca_2_SiO_4_), and zoisite (Ca_2_Al_3_(SiO_4_)_3_(OH)). The phase of PS is active Si-Al and aluminum silicon (Al_1.7_Si_0.15_O_2.85_). The mineral composition of GS is active Si-Al, SiO_2_, and clinoferrosilite (FeSiO_3_). The secondary hydration reaction of CWM is promoted by active Si-Al.

#### 2.1.4. Specific Surface Area

The power supply voltage and temperature range of the Blaine specific surface area tester are 220V ± 10% and 8–34 °C, respectively. Its timing accuracy and measurement accuracy are <0.2 s and <1%, respectively. CFA, RM, BFS, CC, HBFS, PS, and GS were ground in a cement mill for a certain time. Then, the specific surface areas of the raw materials were tested based on the Blaine method of GB 175–2007 [22]. The specific surface areas of CFA, RM, BFS, CC, HBFS, PS, and GS were 525 m^2^/kg, 734 m^2^/kg, 446 m^2^/kg, 378 m^2^/kg, 449 m^2^/kg, 425 m^2^/kg, and 400 m^2^/kg, respectively.

### 2.2. Experimental Design of CWM

The mixing values of the three groups of CWM samples were determined according to the preliminary tests. The performance of the cementitious material is the best in the preliminary test when the mass percentages of CFA, RM, and BFS are 30 wt.%, 10 wt.%, and 20 wt.%, respectively. On the one hand, the dosage of clinker should be reduced as much as possible by increasing the dosage of PS in circulating fluidized bed fly ash-based multi-solid waste cementitious material (CWM). On the other hand, the performance of the CWM should also meet the performance index of 42.5 fly ash Portland cement. At present, many experiments show that the performance of CWM could stably reach the standard of 42.5 fly ash Portland cement when the PS dosage is 10 wt.%. According to the requirements of the GB/T 17671–2021 [23], CWM1, CWM2, and CWM3 were prepared using CFA, RM, BFS, CC, and an admixture (HBFS/PS/GS), as shown in Table 2. The different mass ratios of (T-CaO + Na_2_O)/(SiO_2_ + Al_2_O_3_) ((Ca + Na)/(Si + Al)) of the three CWMs were also calculated, and the mass ratios of (Ca + Na)/(Si + Al) of CWM1, CWM2, and CWM3 were 0.81, 0.84, and 0.78, respectively. The comprehensive comparison of the properties and microstructure of the three CWMs is analysed in the next section based on different mass ratios of (Ca + Na)/(Si + Al).

### 2.3. Preparation of CWM

**Figure 3:**
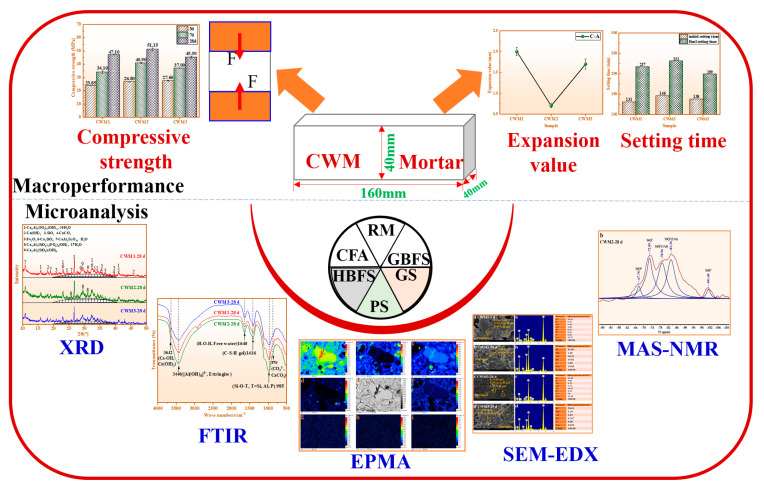
Macro-performance and microanalysis of CWM.

The macro-performance and microanalysis items of CWM samples are summarized in Figure 3. Based on the dosage of the raw materials shown in Table 2, CWM (40 × 40 × 160 mm^3^) was prepared. The raw material and sand are mixed in a mass ratio of 1:3 in the mortar making process. Then, the CWM mortar was cured in a standard curing box with a temperature of 20 ± 1 °C and a relative humidity of 95 ± 1%. Then, the strength value of the CWM was evaluated on the press display. The setting times and volume expansion were checked according to GB 175–2007 [22]. CWM pastes were prepared to detect the microscopic characteristics (XRD, FTIR, MAS-NMR, SEM-EDX, and EPMA).

### 2.4. Test Methods

#### 2.4.1. Performance Test

The compressive strength of the three CWMs was tested with standard press equipment (HYE-300-10) based on the experimental operation of GB/T 17671–2021 [23]. The boiling expansion values of three CWMs were obtained according to GB 175–2007 [22]. The setting time and SO_3_ content of CWMs were tested by the Vicat apparatus and XRF, respectively. In addition, the content of f-CaO and specific surface areas were determined based on EN 451-1-2017 [24] and GB 175–2007 [22], respectively.

#### 2.4.2. Microstructure Analysis

The mineral species and functional groups of CWMs were obtained by XRD (Bruke Company, Karlsruhe, Germany) and Nicolet’s IS10 FTIR (NICOLI, Madison, WI, USA) spectrometers, respectively. Thereinto, the D8 ADVANCE X-ray diffractometer (XRD) is sourced from Bruke Company, Karlsruhe, Germany. Its tube current is 40 mA, tube voltage is 40 kV, and the wavelength of Cu target is 1.5406 angstroms. The polymerization degree and structure of [SiO_4_] in CWMs were tested by a JMM-EC600R nuclear magnetic resonance (JEOL, Tokyo, Japan) spectrometer (^29^Si and ^31^P). The micromorphology and mineral distribution were determined by a SU8020 Gemini cold field scanning electron microscope and energy dispersive X-ray (SEM-EDX) (Hitachi, Tokyo, Japan). Inductively coupled plasma-mass spectrometry (ICP-MS) 7800 (Agilent Corporation, Santa Clara, CA, USA) was used to detect the leaching concentration of harmful elements. The main elements of CWM were quantitatively analysed by JXA-8230 electron probe microanalysis (EPMA) (JEOL, Tokyo, Japan).

## 3. Results and Discussion

### 3.1. Macroperformance of CWM

#### 3.1.1. Compressive Strength

**Figure 4:**
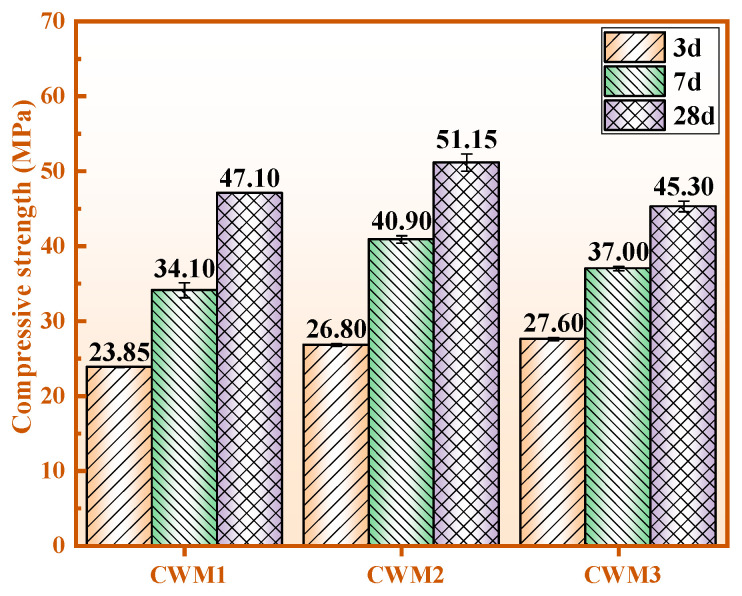
Compressive strength of the three CWMs.

Figure 4 shows the compressive strengths of the three CWMs at 3–28 days. It is obvious from Figure 4 that the compressive strengths of CWM1, CWM2, and CWM3 at 3 days and 28 days all meet the requirements of P. F 42.5. Comprehensive comparison of the three CWMs shows that the compressive strength of CWM2 is optimal, as the mass ratio of (Ca + Na)/(Si + Al) is 0.84. The strength values of CWM2 are 26.80 MPa, 40.90 MPa, and 51.15 MPa at 3 days, 7 days, and 28 days. A possible explanation for this phenomenon is that PS as an admixture is more beneficial to the strength development of the CWM system than HBFS and GS.

#### 3.1.2. Volume Stability and Setting Time

**Figure 5:**
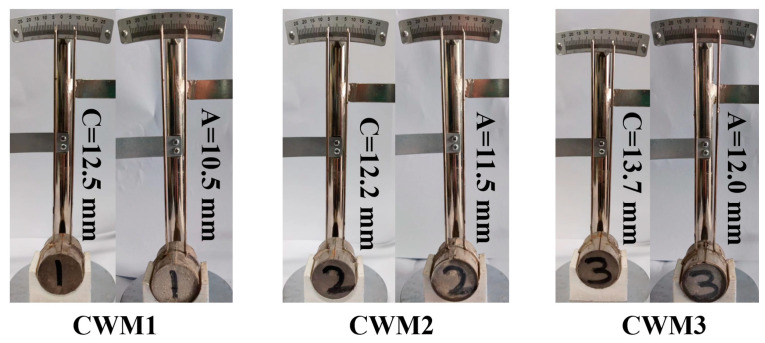
Comparison diagram of volume expansion test results of three CWMs.

**Figure 6:**
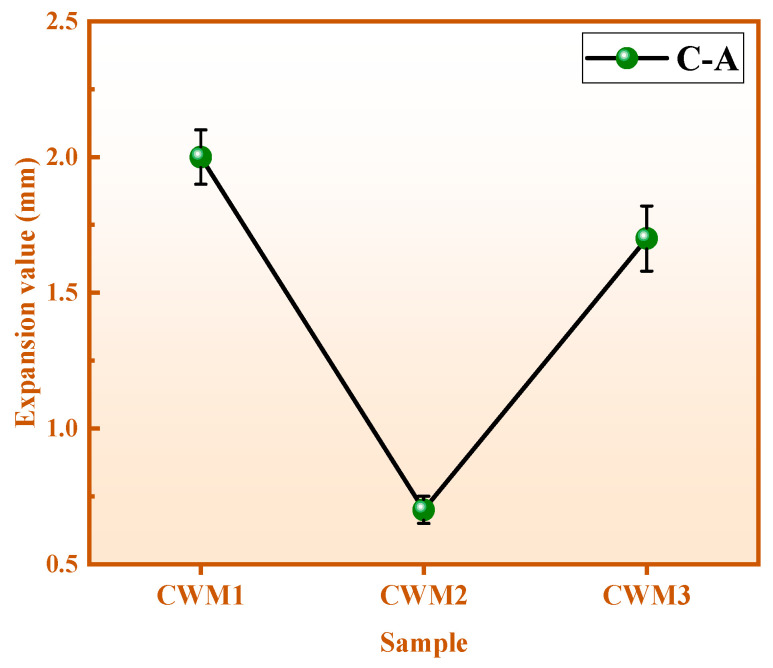
Expansion value of three CWMs.

**Figure 7:**
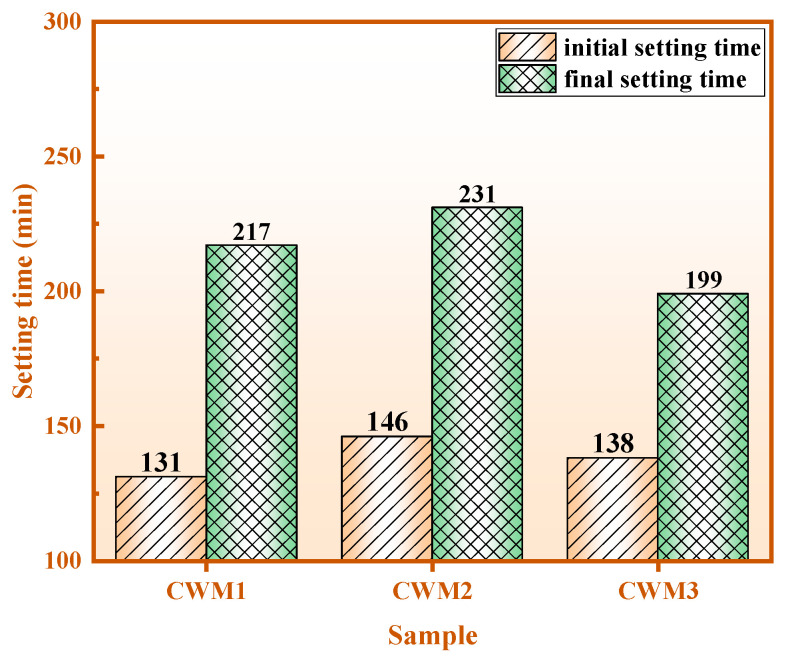
Setting time of three CWMs.

Its strength, volume stability, setting time, and other properties must meet the P. F 42.5 of GB 175–2007 [22] standards because CWM is prepared using CFA, RM, BFS, and an admixture (HBFS/PS/GS). The performance indicators and results are displayed in Figure 5, Figure 6 and Figure 7 and Table 3. It is obvious from Figure 5 that the volume values of CWM samples after boiling (C) in water are higher than those before boiling (A), which means that the volume of the three CWMs is expanded (C-A) under the action of boiling. The expansion value results of the three CWMs are shown in Figure 6. Owing to the comparison of the three CWMs, the volume expansion value of CWM2 is the lowest (0.70 mm) when the mass ratio of (Ca + Na)/(Si + Al) is 0.84. This reason is that the volume stability of CWM2 is optimal under the action of the PS admixture.

The transportation and pouring of CWM are affected by the initial setting time. The CWM hardening and construction progress are affected by the final setting time. Therefore, the initial setting time of CWM should be no less than 45 min based on GB 175–2007, and the final setting time should be no less than 600 min. As illustrated in Figure 7, the setting time of the three CWMs meets GB 175–2007 [22], and the initial setting time and final setting time of CWM2 are 146 min and 231 min, respectively, which are higher than those of the other CWMs. The PS particles are adsorbed on the hydrated product film, then the ions and water are blocked through the film, and the hydration rate is reduced, which eventually leads to an increase in the setting time of the cementitious material [25].

In Table 3, the contents of SO_3_ and MgO in CWM2 were 2.61 wt.% and 2.56 wt.%, which meets the GB 175–2007 requirements of SO_3_ lower than 3.5 wt.% and MgO lower than 6.0 wt.% [22]. Overall, the performance of the three CWMs meets GB 175–2007 [22], and the performance of CWM2 is the relatively optimum one.

### 3.2. Mineral Composition Analysis

**Figure 8:**
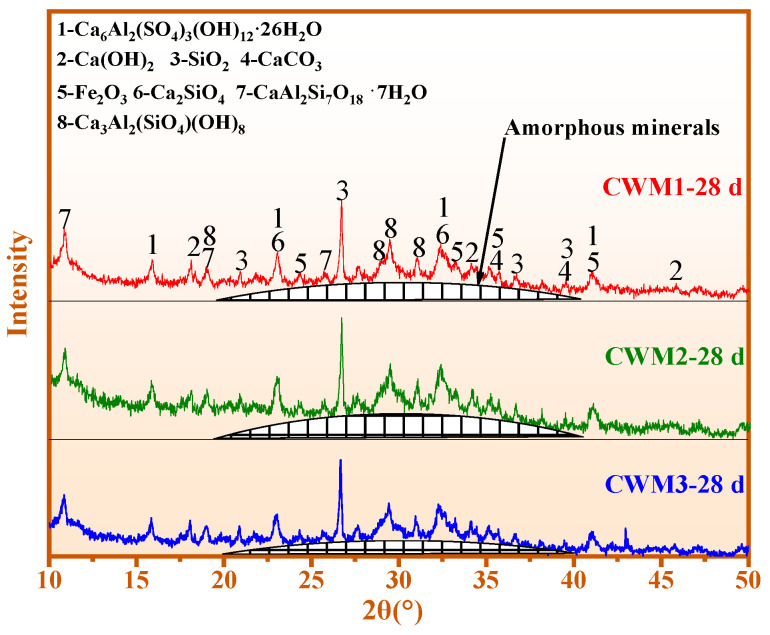
XRD patterns of the three CWMs at 28 days.

Figure 8 shows the phase analysis of CWM1, CWM2, and CWM3 at 28 days. The main minerals of the three CWMs are amorphous minerals, ettringite (Ca_6_Al_2_(SO_4_)_3_(OH)_12_·26H_2_O), portlandite (Ca(OH)_2_), unreacted quartz (SiO_2_), calcite (CaCO_3_), hematite (Fe_2_O_3_), dicalcium silicate (Ca_2_SiO_4_), metaheulandite (CaAl_2_Si_7_O_18_·7H_2_O), and katoite (Ca_3_Al_2_(SiO_4_)(OH)_8_). According to the comprehensive comparison of Figure 8, the strength of amorphous peak in CWM2 is the highest when the mass ratio of (Ca + Na)/(Si + Al) is 0.84, which is consistent with that of ettringite. This result shows that the formation of hydration products in CWM2 is more easily promoted under the action of the PS admixture.

The relevant hydration reactions are analysed as follows. C-S-H gel is formed by the direct reaction of Ca_2_SiO_4_ and Ca_3_SiO_5_ in cement clinker (CC). C-S-H gel is also produced from the reaction of f-CaO in CFA and active SiO_2_ in an alkaline environment (OH^−^). Ettringite is generated from the reaction of CaSO_4_ (SO_3_) in CFA and Ca_3_Al_2_O_6_ in CC. The formation of C/N-A-S-H gel is promoted by active Si-Al of admixture in an alkaline environment (OH^−^). Amorphous minerals could be composed of C-S-H gel and C/N-A-S-H gel in CWM. The relevant hydration mechanism of CWMs is further demonstrated in the following sections.

These related chemical equations are as follows:

(1) $${f\text{-}\mathrm{CaO}+\mathrm{OH}}^{-}{+\mathrm{Active}\;\mathrm{SiO}}_{2}{+H}_{2}O\;\to \;C\text{-}S\text{-}H\;\mathrm{gel}$$

(2) $${\mathrm{CaSO}}_{4}{(\mathrm{SO}}_{3}{)+\mathrm{Ca}}_{3}{\mathrm{Al}}_{2}O_{6}{+H}_{2}O\;\to {\;\mathrm{Ca}}_{6}{\mathrm{Al}}_{2}{{(\mathrm{SO}}_{4})}_{3}{\left(\mathrm{OH}\right)}_{12}{\cdot 26H}_{2}O$$

(3) $${\mathrm{AlO}}_{2}{}^{-}{+\mathrm{OH}}^{-}{+H}_{2}O\;\to {\;[H}_{3}{\mathrm{AlO}}_{4}{]}^{2-}$$

(4) $${\mathrm{SiO}}_{2}{+\mathrm{OH}}^{-}{+H}_{2}O\;\to {\;[H}_{3}{\mathrm{SiO}}_{4}{]}^{-}$$

(5) $${{[H}_{3}{\mathrm{AlO}}_{4}]}^{2-}{+[H}_{3}{\mathrm{SiO}}_{4}{]}^{-}{+\mathrm{Ca}}^{2+}{/\mathrm{Na}}^{+}\;\to \;C/N\text{-}A\text{-}S\text{-}H\cdot \mathrm{gel}$$

**Figure 9:**
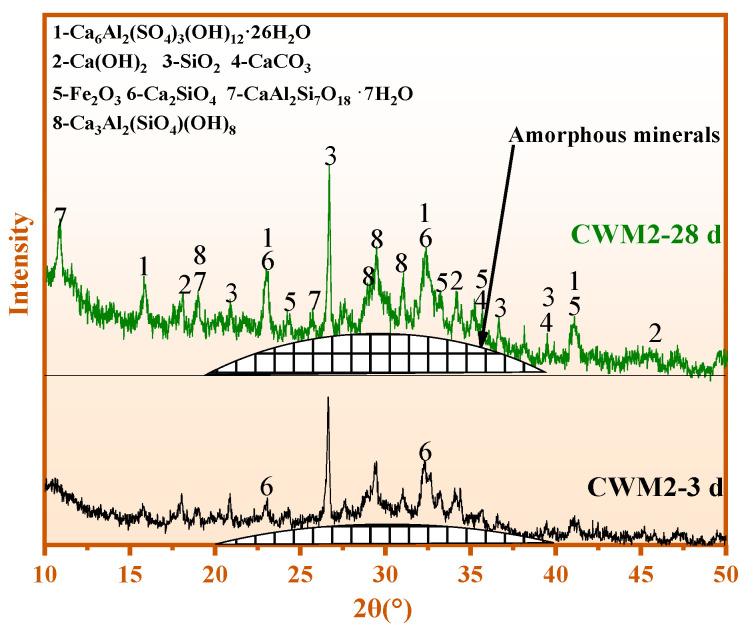
XRD of CWM2 at 3 days and 28 days.

The mineral evolution of CWM2 with hydration time (3 days to 28 days) is shown in Figure 9. As shown in Figure 9, the strength of the amorphous peak in CWM2 rises with hydration time, which is consistent to that of ettringite. However, the intensities of Ca(OH)_2_ show the contrary tendency. The phenomenon indicates that the C-A-S-H gel is formed through the participation of Ca(OH)_2_ in the hydration reaction as the hydration time increases. This result suggests that the production of hydration products is promoted in CWM2 with hydration time, resulting in its performance improvement.

### 3.3. Chemical Bond Analysis

**Figure 10:**
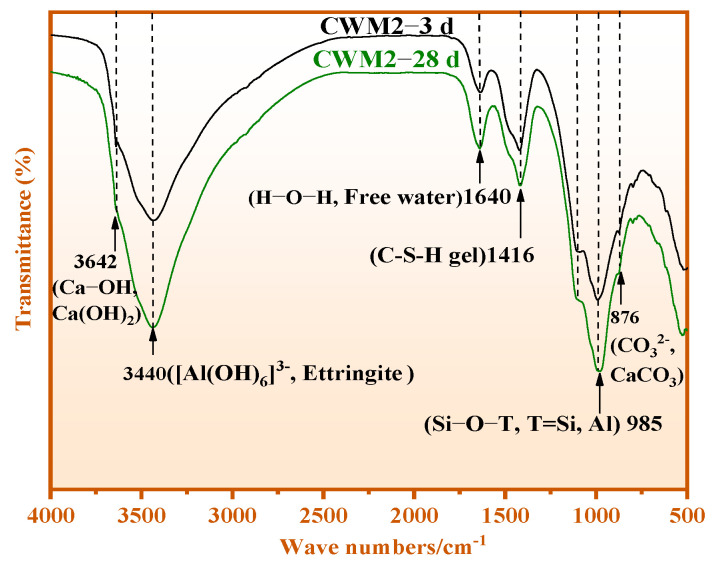
FTIR results of CWM2-3 d and CWM2-28 d.

In Figure 10, the chemical bond analysis of CWM2 at 3–28 days are shown. The bands at 3642 cm^−1^ and 3440 cm^−1^ are attributed to Ca-OH in Ca(OH)_2_ and Al-OH in ettringite, respectively [26,27]. The absorption bands at 1640 cm^−1^ and 1416 cm^−1^ are attributed to H-O-H in free water and Si-OH in the C-S-H gel, respectively [28]. The band at 985 cm^−1^ and 876 cm^−1^ corresponds to Si-O-T (T = Si/Al) within the C/N-A-S-H gel [19,29] and CO_3_^2−^ in CaCO_3_ [30]. It can also be seen that the intensity of bonds at 3642 and 1640 cm^−1^ declined with reaction time. The change rule indicates that Ca(OH)_2_ reacts with active Si-Al in the CWM2 system to produce C-A-S-H gel. Meanwhile, the absorption peak intensities of Al-OH, Si-OH, and Si-O-T rise gradually with hydration time. The reason of these results is that the formation of C-S-H gel, C/N-A-S-H gel, and ettringite is promoted with hydration time under the action of the PS admixture. Therefore, C-S-H gel and C/N-A-S-H gel exist in amorphous minerals of XRD.

**Figure 11:**
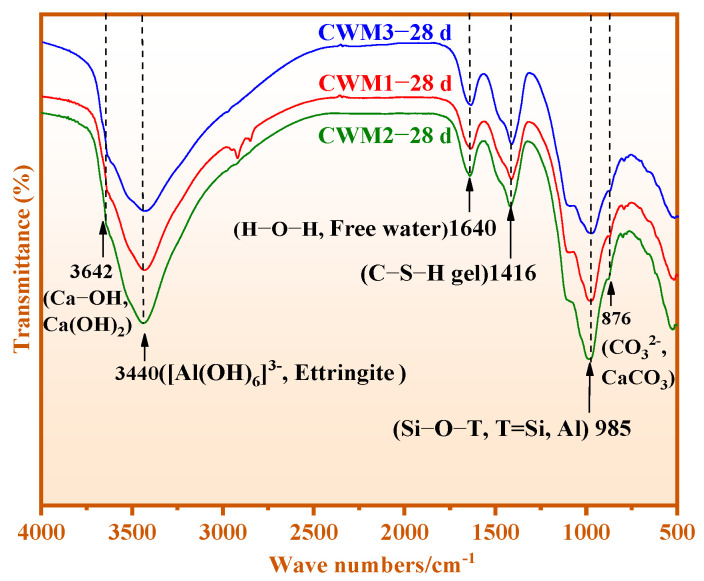
FTIR results of CWM1, CWM2, and CWM3 at 28 days.

Figure 11 shows the chemical bond results of CWM1, CWM2, and CWM3 at 28 days. According to comparison, it is found that the absorption strengths of the [Al(OH)_6_]^3−^ (Al-OH), Si-OH, and Si-O-T bonds in CWM2 are higher than those of other CWMs. This result indicates that more C-S-H gel, C/N-A-S-H gel, and ettringite are generated in CWM2-28 d. The reason is that the formation of more hydration products in the CWM is promoted by the PS admixture compared with HBFS and GS. Thus, the polymerized structure of CWM2 is the highest than others.

### 3.4. Molecular Structure Analysis

The ^29^Si and ^31^P NMR spectra were used to analyse the molecular structure of CWMs. The number of one oxygen atom (adjacent bridge oxygen atom) used by two or more silicon oxygen tetrahedrons [SiO_4_] is presented as SiQ*^n^* (*n* = 0, 1, 2, 3, and 4). Zhang [31] proved that the polymerization degree of the [SiO_4_] structure can be quantitatively analysed using the peak area of relative bridging oxygen (RBO).

The polymerization degree in CWM is calculated by Formula (6):

(6) $$\mathrm{RBO}=\frac{1}{4}(1\times \frac{Q^{1}}{\sum Q^{n}}+2\times \frac{Q^{2}}{\sum Q^{n}}+3\times \frac{Q^{3}}{\sum Q^{n}}+4\times \frac{Q^{4}}{\sum Q^{n}})=\frac{1}{4}\frac{\sum n\cdot Q^{n}}{\sum Q^{n}}$$

where Q*^n^* is the relative peak area of [SiO_4_] with RBO number *n*.

**Figure 12:**
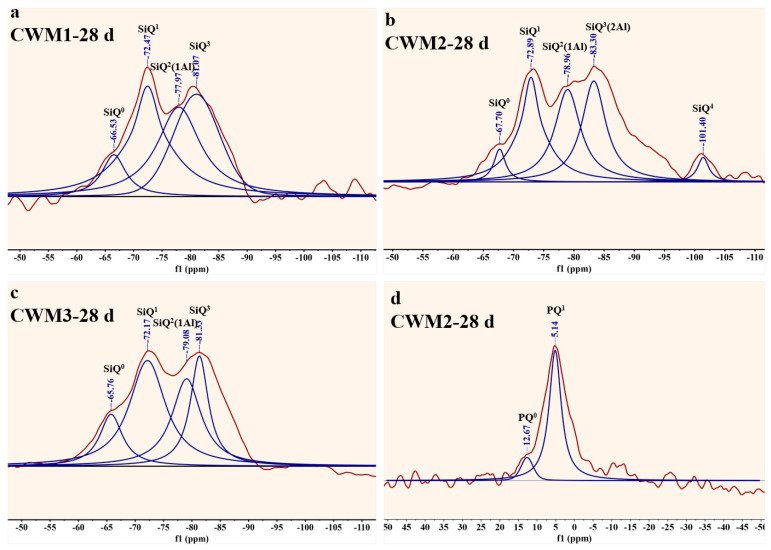
^29^Si NMR and ^31^P NMR data graph of the CWM. (**a**) CWM1-28d, (**b**) CWM2-28 d, (**c**) CWM3-28 d, (**d**) CWM2-28 d (^31^P NMR).

Next, the nuclear magnetic resonance of CWM is analyzed in detail, as follows. The ^29^Si NMR spectra, ^31^P NMR spectra, and polymerization degree are shown in Figure 12 and Table 4, respectively. It is obvious that there are five kinds of molecular structures in the ^29^Si NMR spectrum of CWM: SiQ^0^, SiQ^1^, SiQ^2^(1Al), SiQ^3^(2Al), and SiQ^4^. The relative peak area of each molecular structure was obtained by MestReNova software and is shown in Table 4. Then, the RBO polymerization degree was calculated by the relative peak area and formula (6). SiQ^0^ corresponds to Ca_2_SiO_4_ or Ca_3_SiO_5_ in CWM. SiQ^2^(1Al) or SiQ^3^(2Al) corresponds to [Si(Al)O_4_] in C-A-S-H gel or N-A-S-H gel (C/N-A-S-H gel). SiQ^4^ at −101.40 ppm and PQ^1^ at −5.14 ppm appear together in CWM2-28 d compared with other CWMs. It can be inferred that [PO_4_] and some [SiO_4_] are connected to form Si-O-P bonds in amorphous phosphosilicate minerals [32,33]. Similar to Al^3+^, the Si of [SiO_4_] is replaced by P to form [Si(P)O_4_], and the result is consistent with the study of Liu et al. [34].

In Table 4, the SiQ^2^(1Al) and SiQ^3^(2Al) peak areas of CWM are optimal (95.74 and 97.87, respectively) at CWM2, and the relative peak area of SiQ^0^ is the lowest. Hence, the amount of C-S-H gel and C/N-A-S-H gel is the maximum in CWM2. In addition, CWM2 contains an extra SiQ^4^ and PQ^1^ relative to the other two CWMs, and the polymerization degree of CWM reaches a peak (51.57%) at CWM2. This finding proved that [PO_4_] is more helpful for improving the [SiO_4_] polymerization degree of CWM2. The phenomenon are consistent with the results of Mysen et al. [35]. Therefore, compared with HBFS and GS admixture, the generation of C-S-H gel and C/N-A-S-H gel is more easily improved by the PS admixture. The polymerization structure of CWM2 is ameliorated by above products. In summary, the performance of CWM2 is the best under the higher polymerization degree.

### 3.5. Micromorphology Analysis

**Figure 13:**
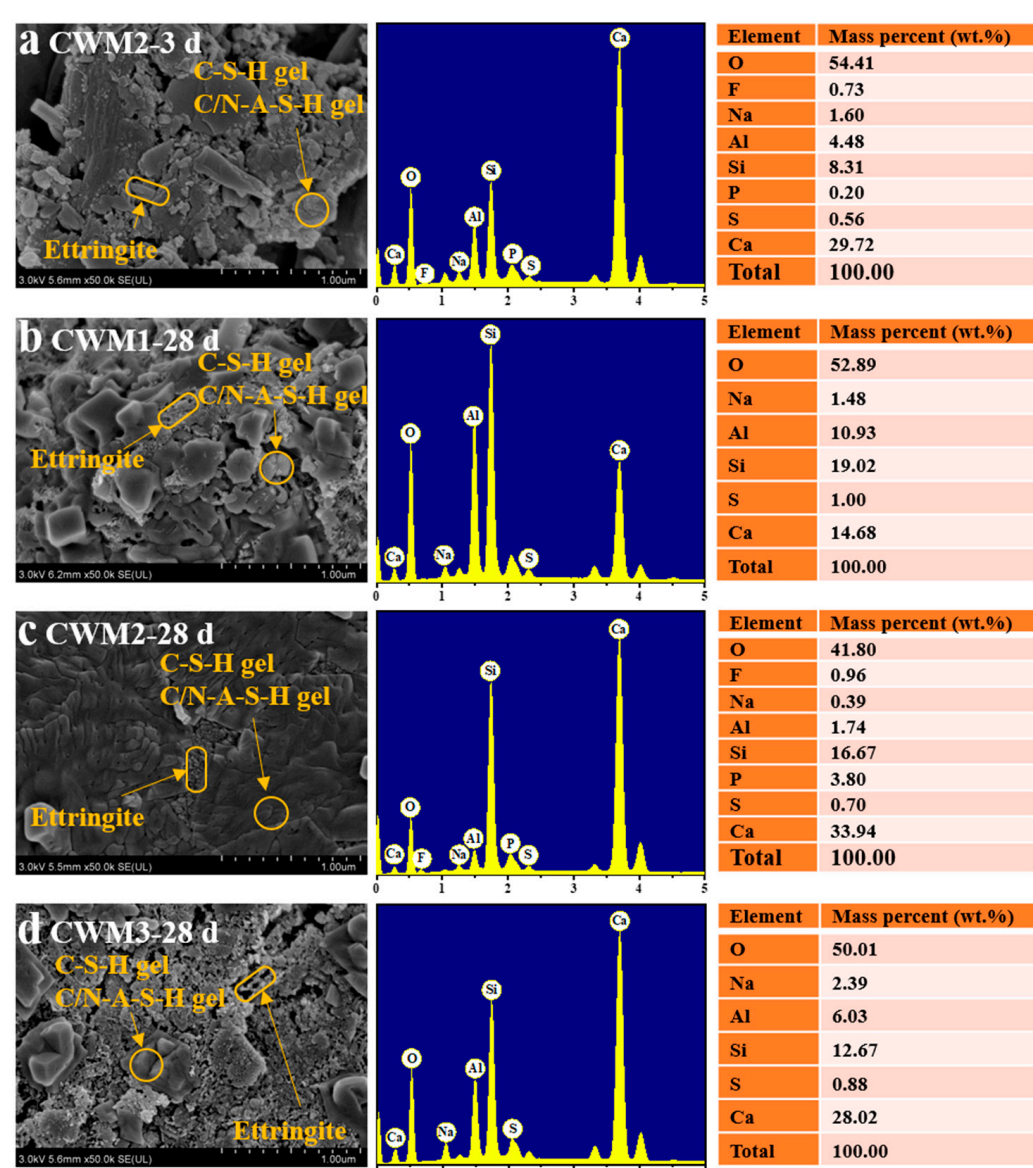
SEM-EDX of (**a**) CWM2-3 d, (**b**) CWM1-28 d, (**c**) CWM2-28 d, and (**d**) CWM3-28 d.

The micromorphology and mineral structure of CWM2-3 d, CWM1-28 d, CWM2-28 d, and CWM3-28 d are provided in Figure 13. Figure 13b shows that the gel products and unreacted minerals are loosely listed together with many pores, and some of the threadiness C-S-H gel, C/N-A-S-H gel, and clavate ettringite are scattered displayed in CWM1. It is possible that the amount of hydration products is relatively low, and the polymerization degree of RBO is low. A similar micromorphology is found in Figure 13d. The overall gel products and minerals are loosely distributed together at CWM3-28 d, and cracks exist in local areas. This phenomenon proves that the densification of the microstructure in CWM3 is lower. In Figure 13c, it is obvious that the micromorphology of the whole matrix of CWM2-28 d is very dense compared with CWM1-28 d and CWM3-28 d. At the same time, the pyknotic micromorphology is shown by C-S-H gels, C/N-A-S-H gels, and clavate ettringite. This can be because the formation of a dense microstructure in CWM2-28 d is promoted by the PS admixture. Moreover, as shown in Figure 13a,c, the microstructure of CWM2 densifies with hydration time, and the number of pores gradually decreases. This result suggests that the compactness of the microstructure is ameliorated with hydration time.

### 3.6. Environmental Performance

#### 3.6.1. Leaching Results

Leaching tests of CFA, RM, PS, GS, CWM1-28 d, CWM2-28 d, and CWM3-28 d are operated based on GB 5086.1-1997. The solid/liquid ratio and the turnover frequency are 0.1 and 32 r/min, respectively (19 h). Inductively coupled plasma-mass spectrometry (ICP-MS) 7800 (Agilent Corporation, Santa Clara, CA, USA) was used to detect the leaching concentration of harmful elements. Then, the leaching concentration of Na, As, Cd, and Hg were measured, as shown in Table 5. It is clear from Table 5 that the leaching concentrations of four harmful elements in CFA and RM surpass the requirements of the WHO drinking water. The leaching results of harmful elements of CWMs are comprehensively compared in Table 5. The leaching concentration of harmful elements in CWM2 is lower than that of other CWMs. The lixiviate values of Na, As, Cd, and Hg in CWM2 are 56.6467, 0.0001, 0.0002, and <0.0001 mg/L, respectively. At the same time, CWM2-28 d meets the WHO requirements for leaching concentrations of Na, As, Cd, and Hg in drinking water. This phenomenon means that the harmful elements of CFA, RM, and PS are well consolidated by CWM2 [27]. These results can be interpreted as the consolidation efficiency of CWM2 being improved under the action of the PS admixture. The relevant consolidation mechanism of harmful elements in CWM2 is analysed in Section 3.6.2.

#### 3.6.2. Consolidation Mechanism

**Figure 14:**
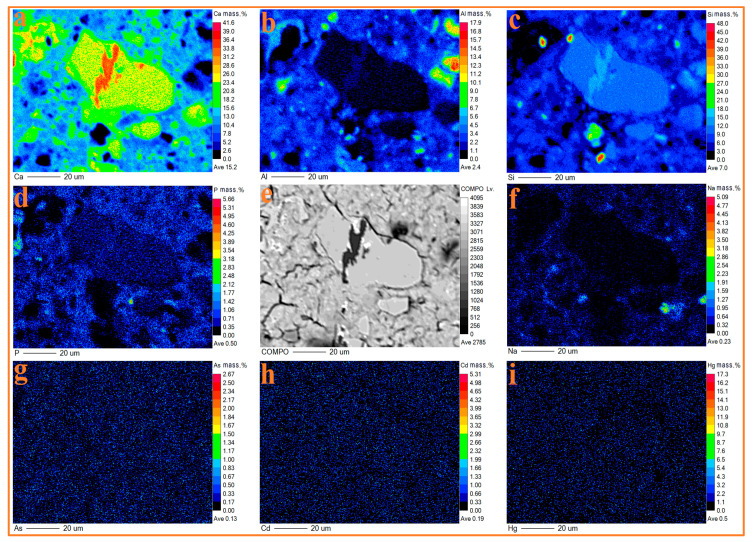
BSE image (**e**) and elemental distribution maps of Ca, Al, Si, P, Na, As, Cd, and Hg (**a**–**d**,**f**–**i**) of CWM20-28 d.

The distribution of Ca, Si, Al, P, Na, As, Cd, and Hg in the CWM2 paste is displayed in Figure 14. As shown in Figure 14, As and Hg are widely distributed in the CWM2 paste at average mass ratios of 0.13 wt.% and 0.50 wt.%, respectively. This is consistent with the theory of Zhang [20] that As and Hg can usually be encapsulated in the circular structure of C-S-H gel. Meanwhile, the distribution of Cd is correlated with the enrichment regions of Ca, Si, and Al. Cd could replace Ca in the C-A-S-H gel or react with Ca on the surface of the C-S-H gel to form minerals containing Ca and Cd [36]. From the distribution of Na shown in Figure 14, it is observed that the distribution of Na is exiguous, and there are still some regions with the enrichment of Na at a content of 0.23 wt.%, where the elements Al and Si are enriched. Na can participate in the secondary hydration reaction to form an N-A-S-H gel [27], which is same with the results of ^29^Si NMR, FTIR, and SEM-EDX. Therefore, the leaching concentrations of Na, As, Pb, and Hg in CWM2 are lower than those in the other CWMs by above consolidation method. CWM2 is proven to be a green cementitious material.

## 4. Conclusions

In this work, the effect of phosphorus slag (PS) admixture on a circulating fluidized bed fly ash (CFA)-based multi-solid waste cementitious material (CWM) system is discussed. The performance, hydration mechanism, and environmental protection of CWM are studied, as follows:

(1) PS as an admixture is more beneficial for improving the performance of CWM systems compared with gasification slag (GS) and blast furnace slag with a high specific surface area (HBFS). The dosage of total solid waste is increased to 70 wt.% in cementitious materials.
(2) The compressive strength of CWM2 is optimal (51.15 MPa) and the expansion value is the lowest (0.70 mm). Moreover, the contents of SO_3_ and MgO are 2.61 wt.% and 2.56 wt.%, respectively, which can reach the level of P. F 42.5. The performances of CWM2 are superior to those of other CWMs under the action of PS admixture.
(3) The main hydration products are C-S-H gel, C/N-A-S-H gel, and ettringite in CWM, which are useful to improve the compactness of the micromorphology. The polymerization degree of CWM2-28 d is the optimal (51.57%) because its polymerization structure is improved by the combination of [PO_4_] and [SiO_4_].
(4) The consolidation capacity of Na, As, Cd, and Hg in CWM2 is higher than that of other CWMs under the action of the PS admixture. The harmful elements of CWM2 are consolidated by physical encapsulation and charge balance, which meet the drinking water requirements of the WHO. Therefore, this work provides a novel idea for the application of PS as an admixture in CFA-based multi-solid waste cementitious material, which is beneficial to the utilization of various solid wastes in building materials.

## Figures and Tables

**Table 1:** Chemical composition of raw materials.

Oxide	CFA	RM	BFS	CC	Admixture		
					HBFS	PS	GS
T-CaO	12.73	21.09	34.14	63.87	39.74	46.67	26.41
f-CaO	4.10	0.00	0.00	0.00	0.00	0.00	0.00
SiO_2_	34.15	19.02	34.64	22.75	29.19	36.79	34.79
Al_2_O_3_	24.04	22.46	18.64	5.76	15.02	2.93	15.96
SO_3_	6.67	0.29	1.66	0.38	2.73	1.30	0.49
Fe_2_O_3_	5.31	15.15	0.86	3.17	0.82	0.13	9.53
MgO	1.25	0.46	6.96	2.06	9.38	1.32	0.98
TiO_2_	0.76	4.33	0.77	0.24	0.81	0.20	0.89
P_2_O_5_	0.23	0.67	0.04	0.19	0.02	3.34	0.07
F	0.00	0.00	0.00	0.00	0.00	3.04	0.00
K_2_O	0.82	0.57	0.63	0.82	0.50	0.69	0.70
Na_2_O	0.20	6.01	0.62	0.33	0.47	0.98	4.89
LOI	10.38	8.84	0.50	0.03	0.60	1.85	3.94
Total	96.54	98.89	99.46	99.6	99.28	99.24	98.65

Note: The loss on ignition (LOI) of CFA, RM, BFS, FBFS, PS, GS, and CC was measured at 800 °C for 4 h.

**Table 2:** Proportion of raw materials (wt.%).

Sample	CFA	RM	BFS	CC	Admixture	(Ca + Na)/(Si + Al) Mass Ratio
CWM1	30	10	20	30	10 (HBFS)	0.81
CWM2	30	10	20	30	10 (PS)	0.84
CWM3	30	10	20	30	10 (GS)	0.78

**Table 3:** Index parameter of CWM (water/cement ratio of 0.37).

NO.	Compressive Strength (MPa)		Setting Time (min)		Expansion Value (mm)	SO_3_ (wt.%)	MgO (wt.%)
	3 Days	28 Days	Initial	Final			
CWM1	23.85	47.10	131	217	2.00	2.75	3.37
CWM2	26.80	51.15	146	231	0.70	2.61	2.56
CWM3	27.60	45.30	138	199	1.70	2.52	2.53
GB 175–2007 [22]	17.00	42.50	≥45	≤600	≤5.00	≤3.50	≤6.00

**Table 4:** NMR data of CWM.

Sample	Peak Position (ppm)	Assign	Relative	Polymerization Degree of RBO
CWM1-28 d	−66.53	SiQ^0^	18.50	46.34%
	−72.47	SiQ^1^	100.00	
	−77.97	SiQ^2^(1Al)	71.68	
	−81.07	SiQ^3^(2Al)	65.32	
CWM2-28 d	−67.7	SiQ^0^	14.89	51.57%
	−72.89	SiQ^1^	100.00	
	−78.96	SiQ^2^(1Al)	95.74	
	−83.30	SiQ^3^(2Al)	97.87	
	−101.40	SiQ^4^	10.64	
CWM3-28 d	−65.76	SiQ^0^	31.78	44.75%
	−72.17	SiQ^1^	100.00	
	−79.08	SiQ^2^(1Al)	68.22	
	−81.33	SiQ^3^(2Al)	53.49	

**Table 5:** Leaching results of harmful elements (mg/L).

Sample	Na	As	Cd	Hg
CFA	5.2753	0.0441	0.0015	<0.0001
RM	685.6372	0.0491	0.0016	0.0022
PS	12.5648	0.0007	<0.0001	<0.0001
GS	37.3006	0.0046	0.0005	<0.0001
CWM1-28 d	70.9931	0.0004	0.0004	0.0002
CWM2-28 d	56.6467	0.0001	0.0002	<0.0001
CWM3-28 d	66.0012	0.0006	0.0004	0.0001
Requirements of the WHO	200.0000	0.0100	0.0030	0.00100

## Data Availability

Data sharing is not applicable to this article.

## References

[1] Jia R., Wang Q., Luo T. (2022). Understanding the workability of alkali-activated phosphorus slag pastes: Effects of alkali dose and silicate modulus on early-age hydration reactions. Cem. Concr. Compos..

[2] Vafaei M., Allahverdi A., Dong P., Bassim N., Mahinroosta M. (2020). Resistance of red clay brick waste/phosphorus slag-based geopolymer mortar to acid solutions of mild concentration. J. Build. Eng..

[3] Wang Y., Xiao R., Hu W., Jiang X., Zhang X., Huang B. (2021). Effect of granulated phosphorus slag on physical, mechanical and microstructural characteristics of Class F fly ash based geopolymer. Constr. Build. Mater..

[4] Hassankhani-Majd Z., Anbia M. (2021). Recovery of valuable materials from phosphorus slag using nitric acid leaching followed by precipitation method. Resour. Conserv. Recycl..

[5] Chen Q., Ding W., Sun H., Peng T. (2019). Mineral carbonation of yellow phosphorus slag and characterization of carbonated product. Energy.

[6] Huang X.-F., Yang X.-B., Zhao D., Ma L.-P., Bao Y.-Z. (2022). Effect of BaF2 and CaF2 on properties of Tb3+-doped yellow phosphorus slag luminescent glass-ceramics. Opt. Mater..

[7] Yu H., Zhu X., Qian G., Gong X., Nie X. (2020). Evaluation of phosphorus slag (PS) content and particle size on the performance modification effect of asphalt. Constr. Build. Mater..

[8] Li X., Zhang Q. (2022). Influence behavior of phosphorus slag and fly ash on the interface transition zone in concrete prepared by cement-red mud. J. Build. Eng..

[9] Mehdizadeh H., Shao X., Mo K.H., Ling T.-C. (2022). Enhancement of early age cementitious properties of yellow phosphorus slag via CO2 aqueous carbonation. Cem. Concr. Compos..

[10] Zhang Z., Wang Q., Yang J. (2017). Hydration mechanisms of composite binders containing phosphorus slag at different temperatures. Constr. Build. Mater..

[11] Liu Z., Li S., Li L., Wang J., Zhou Y., Wang D. (2019). One-step high efficiency crystallization of zeolite A from ultra-fine circulating fluidized bed fly ash by hydrothermal synthesis method. Fuel.

[12] Zhang W., Wang S., Ran J., Lin H., Kang W., Zhu J. (2022). Research progress on the performance of circulating fluidized bed combustion ash and its utilization in China. J. Build. Eng..

[13] He P., Zhang X., Chen H., Zhang Y. (2021). Waste-to-resource strategies for the use of circulating fluidized bed fly ash in construction materials: A mini review. Powder Technol..

[14] Liang G., Li H., Zhu H., Liu T., Chen Q., Guo H. (2021). Reuse of waste glass powder in alkali-activated metakaolin/fly ash pastes: Physical properties, reaction kinetics and microstructure. Resour. Conserv. Recycl..

[15] Li D., Sun R., Wang D., Ren C., Fang K. (2021). Study on the pozzolanic activity of ultrafine circulating fluidized-bed fly ash prepared by jet mill. Fuel.

[16] Li D., Wang D., Ren C., Rui Y. (2018). Investigation of rheological properties of fresh cement paste containing ultrafine circulating fluidized bed fly ash. Constr. Build. Mater..

[17] Zahedi M., Jafari K., Rajabipour F. (2020). Properties and durability of concrete containing fluidized bed combustion (FBC) fly ash. Constr. Build. Mater..

[18] Chen X., Gao J., Yan Y., Liu Y. (2017). Investigation of expansion properties of cement paste with circulating fluidized bed fly ash. Constr. Build. Mater..

[19] Zhang W., Liu X., Zhang Z., Li Y., Gu J. (2022). Synergic effects of circulating fluidized bed fly ash-red mud-blast furnace slag in green cementitious materials: Hydration products and environmental performance. J. Build. Eng..

[20] Zhang W., Liu X., Zhang Z., Li Y., Gu J., Wang Y., Xue Y. (2022). Circulating fluidized bed fly ash-blast furnace slag based cementitious materials: Hydration behaviors and performance. Constr. Build. Mater..

[21] Zhang N., Tang B., Liu X. (2021). Cementitious activity of iron ore tailing and its utilization in cementitious materials, bricks and concrete. Constr. Build. Mater..

[22] (2007). GB 175-2007, Common Portland Cement. https://www.antpedia.com/standard/5156435.html.

[23] (2021). GB/T 17671-2021, Test Method of Cement Mortar Strength (IOS Method). http://www.doc88.com/p-08773985826297.html.

[24] (2017). EN 451-1-2017, Method of Testing Fly Ash—Part 1: Determination of Free Calcium Oxide Content. https://www.doc88.com/p-9189147009468.html.

[25] Wu Y., Zhang Q., Li L. (2019). Effect of phosphorus slag on retarding properties of ordinary portland cement. Bull. Chin. Ceram. Soc..

[26] Zhang W., Gu J., Zhou X., Li Y., Wang Y., Xue Y., Liu X. (2021). Circulating fluidized bed fly ash based multi-solid wastes road base materials: Hydration characteristics and utilization of SO_3_ and f-CaO. J. Clean. Prod..

[27] Zhang W., Liu X., Wang Y., Li Z., Li Y., Ren Y. (2021). Binary reaction behaviors of red mud based cementitious material: Hydration characteristics and Na^+^ utilization. J. Hazard. Mater..

[28] Liu X., Zhao X., Yin H., Chen J., Zhang N. (2018). Intermediate-calcium based cementitious materials prepared by MSWI fly ash and other solid wastes: Hydration characteristics and heavy metals solidification behavior. J. Hazard. Mater..

[29] Liu X., Yang S., Liu S., Yang Y. (2021). Performance and mechanism of phosphorus removal by slag ceramsite filler. Process Saf. Environ. Prot..

[30] Hao X., Liu X., Zhang Z., Zhang W., Lu Y., Wang Y., Yang T. (2022). In-depth insight into the cementitious synergistic effect of steel slag and red mud on the properties of composite cementitious materials. J. Build. Eng..

[31] Zhang J., Sun H., Sun Y., Zhang N. (2009). Correlation between ~(29)Si polymerization and cementitious activity of coal gangue. J. Zhejiang Univ..

[32] Szumera M. (2014). Structural investigations of silicate–phosphate glasses containing MoO_3_ by FTIR, Raman and 31P MAS NMR spectroscopies. Spectrochim. Acta Part A Mol. Biomol. Spectrosc..

[33] Cody G.D., Mysen B., Sághi-Szabó G., A Tossell J. (2001). Silicate-phosphate interactions in silicate glasses and melts: I. A multinuclear (27Al,29Si,31P) MAS NMR and ab initio chemical shielding (31P) study of phosphorous speciation in silicate glasses. Geochim. Cosmochim. Acta.

[34] Liu C., Zhang R., Zhao X., Jia J., Min Y. (2021). Quantification of phosphorus structures in CaO–SiO_2_–P_2_O_5_ glasses via Raman spectroscopy. J. Non-Cryst. Solids.

[35] Mysen B.O., Ryerson F., Virgo D. (1981). The structural role of phosphorus in silicate melts. Am. Mineral..

[36] Liu M., Zhao Y., Yu Z., Cao Z. (2021). Impact of Ni(Ⅱ) and Cd(Ⅱ) on the hydration and microstructure of cement pastes for immobilization: C-A-S-H composition and binding characteristic. Constr. Build. Mater..

